# Synthesis of Manganese Oxide Sorbent for the Extraction of Lithium from Hydromineral Raw Materials

**DOI:** 10.3390/ma16247548

**Published:** 2023-12-07

**Authors:** Zaure Karshyga, Albina Yersaiynova, Azamat Yessengaziyev, Bauyrzhan Orynbayev, Marina Kvyatkovskaya, Igor Silachyov

**Affiliations:** 1 The Institute of Metallurgy and Ore Beneficiation, Satbayev University, Almaty 050013, Kazakhstan; z.karshyga@satbayev.university (Z.K.); a.yessengaziyev@satbayev.university (A.Y.); bauyrzhan.orynbayev@stud.satbayev.university (B.O.); kmn_55@mail.ru (M.K.); 2 The Institute of Nuclear Physics, Almaty 050032, Kazakhstan; silachyov@inp.kz

**Keywords:** brines, lithium, synthesis, calcination, precursor, sorbents

## Abstract

The article presents the research results for the synthesis of inorganic sorbents based on manganese oxide compounds. It shows the results of the lithium sorption from brines with the use of synthesized sorbents. The effect of temperature, the molar ratio of Li/Mn, and the duration for obtaining a lithium-manganese precursor and its acid treatment was studied. The sorption characteristics of the synthesized sorbents were studied. The effect of the ratio of the sorbent mass to the brine volume and the duration of the process on the sorption of lithium from brine were studied. In this case, the sorbent recovery of lithium was ~86%. A kinetic model of the lithium sorption from brine on a synthesized sorbent was determined. The kinetics of the lithium sorption was described by a pseudo-second-order model, which implies limiting the speed of the process due to a chemical reaction.

## 1. Introduction

Lithium is one of the most important energy materials and strategic resources of the 21st century. It is represented in high technologies covering many areas of human activity. Lithium has become extremely important in the production of rechargeable lithium-ion batteries (LIBs), which have revolutionized the market supply and demand of renewable energy due to their unique technical characteristics (specific energy density 100–265 Wh/kg, specific power 250–340 Wh/kg, service life 400–1200 cycles) [1,2]. LIBs are used in smartphones, computers, hybrid cars, and electric vehicles. Besides batteries, lithium has large areas of application in the production of glass and ceramics (30%), lubricants (11%), metallurgy (4%), as well as in the production of chemicals, pharmaceuticals, and rubbers [3].

The created high global demand for this metal contributes to research and the search for technological solutions involving the processing of lithium-containing hydromineral raw materials, including associated reservoir brines.

Currently, the use of natural mineral raw materials is proposed to recover valuable metals from various hydromineral sources and industrial solutions [4,5]. In [6], methods intended to modify natural aluminosilicate and carbon-mineral sorbents were used to increase their sorption capacity.

According to the literature, such methods as natural evaporation, deposition, electrolysis, and others are used to process lithium raw materials [7,8,9,10,11]. In [12], a method of evaporation and crystallization was proposed to process solutions. In [13], precipitation methods were used to recover lithium from brines. In [14,15,16,17], ion exchange and extraction methods along with a combination of these methods with precipitation [18] were used for brines containing high concentrations of calcium and magnesium. There are known methods for the sorption extraction of lithium from sea water and brines with the use of spinel-type manganese oxide; aluminum compounds have an extremely high selectivity for the extraction of lithium from sea water [19,20,21,22]. These materials have a high adsorption capacity; lithium was concentrated more than 400 times in alkaline media (pH ~8). 

Sorbents obtained based on double compounds of aluminum and lithium LiCl·2Al(OH)_3_·mH_2_O (DHAL-Cl) have high performance and are stable in brines with low pH [23,24,25,26]. The interaction occurs during the crystallization of DGAL-Cl via an intercalation mechanism with the introduction of Li+ cations and Cl¯ anions into the interlayer space. In this case, an intermediate phase of aluminum hydroxide with a deformed structure is formed. It is preserved during complete or partial deintercalation of lithium chloride from DGAL-Cl and is characterized by increased reactivity. Defective DGAL-Cl with a lithium deficiency in its composition is a sorbent selective for lithium. However, the deposition of impurities and mechanical inclusions on it can result in a narrowing of transport channels and to undersaturation of the sorbent with lithium during repeated long-term operation of the sorbent in sorption–desorption cycles under dynamic conditions, and as a result, the lithium deficiency in the sorbent may increase above the required limits after desorption [27]. The lithium deficiency should not exceed 35% of its total content in it to avoid destruction of the sorbent. It determines, and thereby limits, the value of the total exchange capacity of 7 mg/g. Effective use of the sorbent requires strict adherence to technological regimes. 

Recently, technology with the use of lithium-ion sieves (LIS) has become one of the most promising for the extraction of lithium from brines and seawater. LIS make it possible to recover lithium with high selectivity from complex solutions with a high content of accompanying components.

In general, LIS are divided into two types according to the chemical composition: type of oxide lithium and manganese (LMO) and type of oxide lithium and titanium (LTO). 

Lithium-ion sieves based on titanium oxides are currently produced in two categories: H_2_TiO_3_ with a layered structure and H_4_Ti_5_O_12_ with spinel structure. When sorbents based on titanium H_2_TiO_3_ synthesized with the sol–gel method by the interaction of CH_3_COOLi and Ti(OC_4_H_9_)_4_, are used, 31.2 mg/g of lithium can be adsorbed [28]. During sorption by a sorbent from a TiO_2_ nanotube with a diameter of 50–70 nm and a length of 1–2 μm, synthesized with a soft hydrothermal method at 150 °C for 48 h, 39.4 mg/g of lithium can be adsorbed from a solution with a concentration of 120 mg/L at alkaline pH [29]. Studies [30] on the adsorption of lithium on various titanium oxides showed that the Li_2_TiO_3_ structure obtained from anatase was more suitable for lithium recovery than that obtained from rutile. However, titanium oxide-based LISs have limited application in recovery of lithium from aqueous solution by applying electrical potential that may hinder future industrial applications. 

Lithium-ion oxides based on spinel-type manganese oxide are currently the most popular selective sorbents. The formation of a three-dimensional structure with lithium as the LiMn_2_O_4_ compound favors the sorption mechanism instead of the two-dimensional layered crystal structure of LiMnO_2_. The smaller size of lithium ions compared to any other alkali metals contributes to the formation of a stable structure of LiMn_2_O_4_, while lithium in LiMnO_2_ occupies the interlayer octahedral region [20,31]. Chitrakar et al. [32] synthesized low-crystalline orthorhombic LiMnO_2_ by the interaction of γ-MnOOH or Mn_2_O_3_ with LiOH∙H_2_O in the solid phase in a steam atmosphere at 120 °C with subsequent heating of samples at 400 °C in an air atmosphere for 4 h to form the cubic structure pf Li_1.6_Mn_1.6_O_4_. After acid treatment of the precursor, the lithium capacity of the resulting sorbent was 33 mg/g. In another study [33], manganese oxide adsorbent H_1.6_Mn_1.6_O_4_ was obtained from Li_1.6_Mn_1.6_O_4_ precursor prepared by calcination of LiMnO_2_ at 400 °C. In this case, two different methods were used for the synthesis of LiMnO_2_, hydrothermal and reflux; the lithium capacity of the resulting sorbents was 40.9 mg/g and 34.1 mg/g, respectively. In [34], Li_0.15_H_0.76_Mg_0.40_–Mn^III^_0.08_Mn^IV^_1.59_O_4_ adsorbent was studied. The adsorbent showed a maximum lithium adsorption capacity of 23 mg/g at pH 6.5. After adsorption, lithium can be desorbed by dilute HCl solution, and the adsorption efficiency of the sample does not decrease even after 10 cycles. 

As the literature data show, the synthesis of LIS based on manganese oxide consists of several stages: preparation of the precursor, its calcination, and acid treatment of the precursor to obtain a sorbent. The decisive role may be played by the first stage of obtaining lithium-manganese oxide, which ensures good contact of the reacting substances with the use of the lithium reagent in the quantities required for the reaction. At the same time, the remaining stages of sorbent synthesis are also important. Therefore, to study all stages of sorbent preparation under various conditions, temperature, duration, etc. are of interest. 

## 2. Materials and Methods

Materials: Lithium hydroxide single-mode LiOH·H_2_O brand “puriss.”; salt acid HCl qualification “puriss.”; Mn_2_O_3_ “puriss. spec.”; MnO “puriss.”.

The object of this study is the formation brines of oil and gas fields of JSC “Mangystaumunaigas” (Kazakhstan) of the following composition, mg/L: 5.9–7.8 Li; 25,000–3,0000 Na; 4000–6000 Ca; 20–670 K; 1200–2000 Mg; 10–700 Fe; 160–320 Sr; 13–14 B; 42,000–60,000 Cl^−^; 670–780 ${SO}_{4}^{2-}$. 

Analysis methods: the quantitative content of basic elements in precursors and sorbents was determined on an atomic emission spectrometer with inductively coupled plasma Optima 8300DV (Perkin Elmer Inc., Waltham, MA, USA). X-ray phase analysis (XRD) was carried out on a diffractometer D8 ADVANCE “BRUKER AXS GmbH”, (Karsruhe, Germany) radiation Cu-Kα, database PDF-2 International Center for Diffraction Data ICDD (Swarthmore, PA, USA).

Thermal analysis of the lithium-manganese oxide sample was performed using an STA 449 F3 Jupiter simultaneous (NETZSCH, Selb, Germany) thermal analysis device. Before heating, the furnace space was evacuated (the percentage of the evacuated volume was ~92%) and then purged with inert gas for 5 min. Heating was carried out at a speed of 10 °C/min. in an atmosphere of highly purified argon. The total volume of incoming gas was maintained within 120 mL/min. The results obtained with the STA 449 F3 Jupiter (NETZSCH, Selb, Germany) were processed with the use of the NETZSCH Proteus software, version 5.1. 

Experimental procedure: Reagents were taken only in the required quantities, according to the given molar ratio; lithium hydroxide LiOH∙H_2_O was dissolved in 100–150 mL of hot distilled water, and then mixed in a porcelain cup with samples of manganese oxides Mn_2_O_3_ and MnO, taken in accordance with the stoichiometry of the reaction of the formation of lithium-manganese oxides and the specified molar ratios Li/Mn. The resulting mixture was kept in a drying cabinet with heating to a set temperature and kept for a set time while stirring and keeping wet, and then the sample was dried until moisture was completely removed. In this way, uniform mixing and contact of all components of the reaction mixture were achieved during interaction with a liquid solution of the lithium hydroxide reagent. After evaporation and drying, this mixture was calcined. After drying, the resulting lithium-manganese oxides (LMOs) were calcined in a muffle furnace with heating to a given temperature and held for a given time. The resulting LMO and calcined precursors were analyzed for lithium and manganese content, and the phase composition was determined.

Precursors were poured with the required amount of dilute hydrochloric acid solution according to the experimental procedure for acid treatment. The process was performed at a given temperature and contact time under stirring in a 3 dm^3^ sealed thermostated cell equipped with a VELP Scientifica LS F201A0151 mechanical stirrer (Usmate Velate, Italy), providing a fixed speed. Constant temperature was maintained using an Aizkraukles TW 2.02 water bath thermostat (ELMI, Riga, Latvia).

The resulting sorbents were washed with distilled water to pH = 6–7 and dried in air at room temperature. The resulting sorbents were analyzed for lithium content, and the phase composition was determined. The filtrates were analyzed for lithium and manganese content.

Sorption was performed under static conditions on an orbital shaker with a rotation of 200 rpm. A given amount of sorbent was placed in 300 cm^3^ dry flasks filled with a given volume of brine, set to a given temperature, and stirred for a certain time to perform sorption. The solution was separated from the sorbent by filtration after sorption. Sorption filtrates were analyzed for lithium, sodium, potassium, iron, calcium, and magnesium content.

The study of sorption kinetics was performed under static conditions on an orbital shaker at a rotation of 200 rpm. To carry out sorption, 0.2 g of the sorbent was placed in dry flasks with a volume of 300 cm^3^, filled with a brine volume of 130 cm^3^, a set temperature was established, and stirred for a certain time. After sorption, the solution was separated from the sorbent by filtration. Sorption filtrates were analyzed for lithium content.

Static exchange capacity, distribution coefficients K_d_, and partition coefficients K_s_ were determined by the Formulas (1)–(3).

Static exchange capacity is calculated by the formula:

(1) $$SEC=\frac{\left(C_{0}-C_{e}\right)\cdot V}{m},$$

where C_0_ is metal concentration in initial solution, mg/dm^3^; C_e_ is residual equilibrium concentration of metal in solution, mg/dm^3^; V—volume of solution, dm^3^; m—mass of dry sorbent, g.

The distribution coefficients K_d_ and partition coefficients K_s_ were determined by the following formulas:

(2) $$K_{d}=\frac{\left(C_{0}-C_{e}\right)\cdot V^{\prime }}{C_{e}\cdot m},$$

where C_0_ is concentration of metal in initial brine, mg/dm^3^; C_e_ is residual equilibrium concentration of metal in solution, mg/dm^3^; V′ is volume of solution, cm^3^; m is mass of dry sorbent, g.

(3) $$K_{s}=\frac{K_{d}^{Li}}{K_{d}^{Me}}$$

where Me is Ca, Mg, Na, K, Fe.

## 3. Results and Discussion

### 3.1. Study of Conditions for Production of Lithium-Manganese Precursors

#### 3.1.1. Preparation of Lithium-Manganese Oxides

The main purpose of the research is to obtain lithium-manganese oxide with the main phase consisting more preferably of LiMnO_2_, LiMn_2_O_4_ compounds.

The interaction of reacting substances can presumably take place in accordance with the following reactions:

Mn_2_O_3_ + MnO + 3 LiOH∙H_2_O + 0.25 O_2_ = 3 LiMnO_2_ + 4.5 H_2_O↑ (4)

2 Mn_2_O_3_ + 2 MnO + 3 LiOH∙H_2_O + 1.25 O_2_ = 3 LiMn_2_O_4_ + 4.5 H_2_O↑ (5)

Study of the temperature effect. The experiments were carried out under the following conditions: temperature—125, 150, 175, and 200 °C, duration—13 h, the mass ratio of manganese oxides to lithium hydroxide monohydrate was taken from the calculation of Li/Mn molar ratio maintenance = 1.

**Figure 1:**
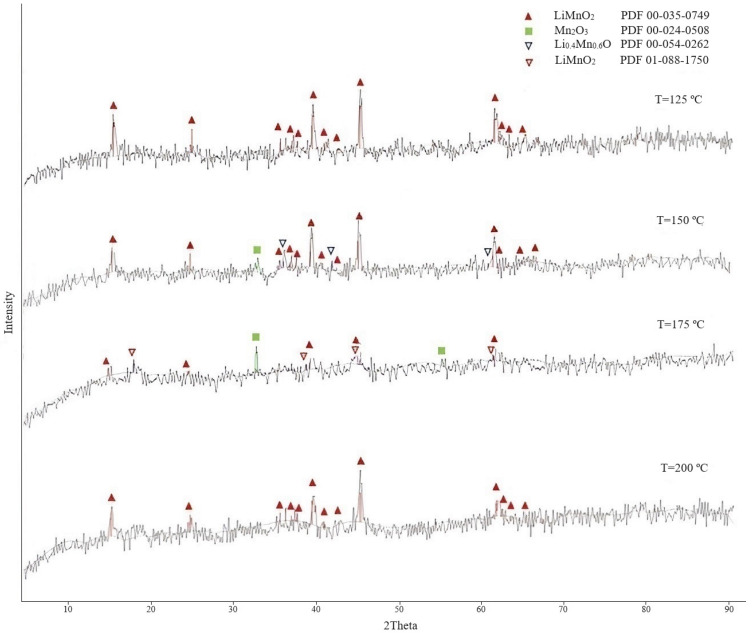
Diffractograms of LMOs obtained with exposure at different temperatures (LiMnO_2_ triangle orthorhombic; LiMnO_2_ rhombus—tetragonal).

The obtained LMOs were studied using the XRD analysis. The results of the XRD analysis are presented in Figure 1.

As the results of studies show, at temperatures of 125 and 200 °C, the reaction of the interaction of manganese oxide with lithium hydroxide fully proceeded with the formation of lithium-manganese oxide LiMnO_2_ with the orthorhombic structure of the crystal lattice (Figure 1). It should be noted that the XRD analysis of samples obtained at 150 and 175 °C showed the presence of the Mn_2_O_3_ phase in the samples; however, this phase was absent in the sample processed at 125 °C. Presumably, the manganese oxide phase was present in the sample obtained at 125 °C in an X-ray amorphous or amorphous state, and the XRD analysis could not identify it. In the sample at 150 °C, there was a Li_0.4_Mn_0.6_O phase with a spinel structure similar in composition to the LiMnO_2_ phase, which presumably may indicate an intermediate stage of LMO formation. At a temperature of 200 °C, only the phase of orthorhombic LiMnO_2_ was identified, as at a temperature of 125 °C. Research results show that a temperature of 125 °C was sufficient to form the lithium-manganese oxide phase LiMnO_2_.

**Figure 2:**
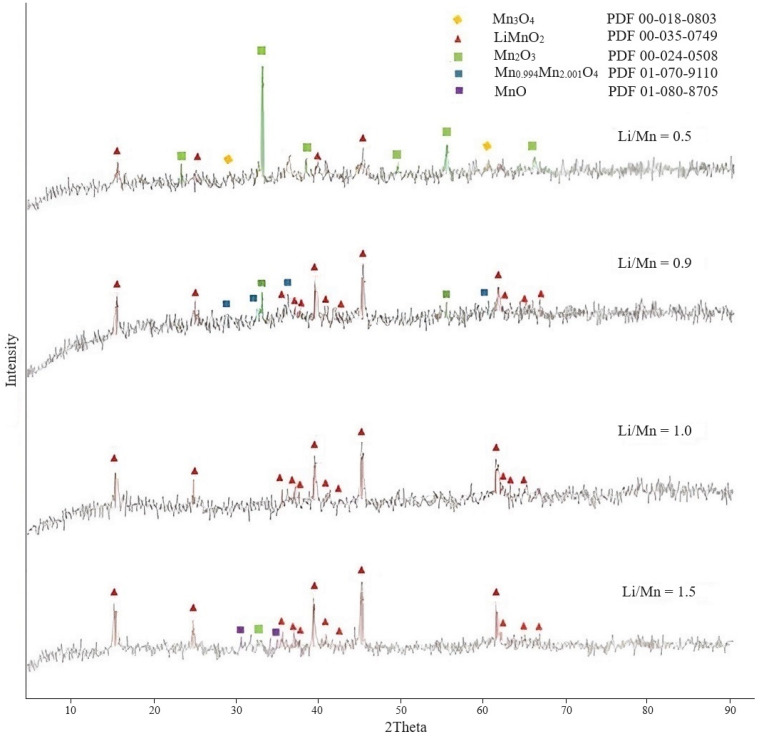
Diffractograms of LMOs obtained at different Li/Mn molar ratios.

Study of the effect of Li/Mn molar ratio. The experiments were performed under the following conditions: the ratio of the mass of manganese oxides to lithium hydroxide monohydrate was taken based on the calculation to maintain the molar ratios Li/Mn = 0.5, 0.9, 1, and 1.5; temperature 125 °C, duration—13 h. The phase composition of the obtained LMOs was studied using X-ray phase analysis. The XRD patterns of lithium-manganese oxides are presented in Figure 2.

The diffractogram of the sample with a molar ratio of Li/Mn = 0.5 showed that the while the process of LiMnO_2_ formation was at an initial stage, the initial manganese oxides were largely present in the sample. With a molar ratio of Li/Mn = 0.9, the process of LMO formation was more active, as indicated by the presence of peaks on the diffractogram corresponding to the LiMnO_2_ phase with a higher intensity. However, the sample also contained phases of the initial manganese oxides that did not react with lithium hydroxide. The XRD diffractogram of the sample substance with a molar ratio Li/Mn = 1 showed that it was represented by the orthorhombic LiMnO_2_ phase, indicating the most complete passage of the process. With a molar ratio of Li/Mn equal to 1.5, lithium-manganese oxide was also actively formed. However, despite the increase in the intensity of LMO peaks, the diffractogram indicateed the presence of a small amount of initial manganese oxides (II), (III). At the same time, in [35], when calcining a mixture of the initial lithium carbonate or hydroxide with manganese dioxide or carbonate at molar ratios Li/Mn 0.75 and 1, the presence of the initial tetragonal MnO_2_ was observed, and when increased to 1.5, the intensity of the spinel peak gradually decreased to an amorphous phase with low crystallinity. In [36], a sorbent prepared from a Li_2_MnO_3_ precursor with a monoclinic structure at a molar ratio of Li/Mn = 2 showed an inability to sorb lithium from a solution (−21.1 mg/g), while a sorbent prepared at a molar ratio of Li/Mn = 1 showed the highest capacity, which was 6.6 mg/g of lithium.

According to the research results, the optimal molar ratio of Li/Mn in the reaction mixture was equal to 1, which was characterized by a more complete interaction of manganese oxides with lithium hydroxide and the formation of LiMnO_2_.

**Figure 3:**
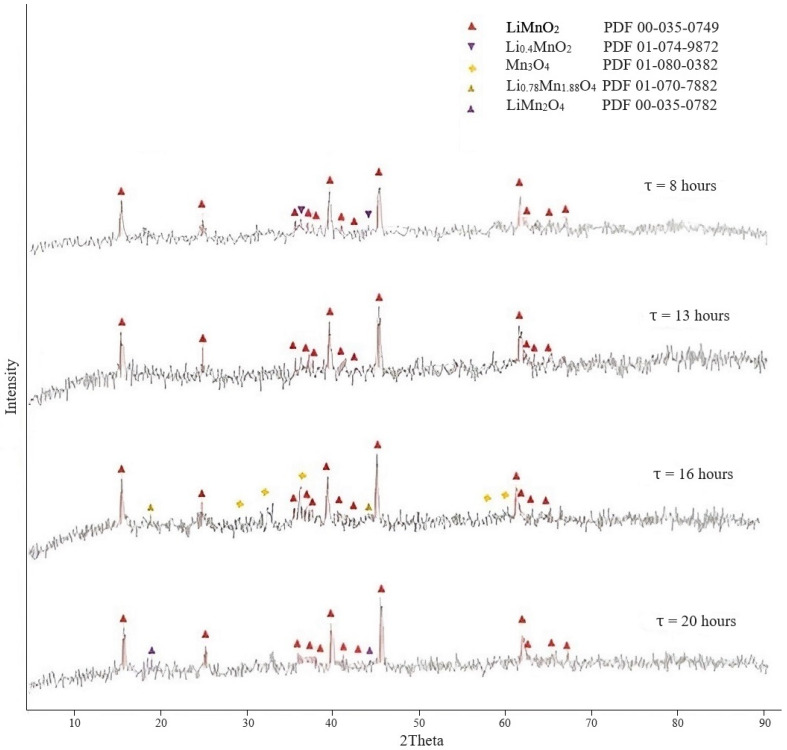
Diffractograms of LMOs obtained at different dwell times.

Study of the effect of the process duration. The experiments were performed under the following conditions: the ratio of the mass of manganese oxides to lithium hydroxide monohydrate was taken based on the calculation to maintain the molar ratio Li/Mn = 1; temperature 125 °C, duration 8, 13, 16, and 20 h. The XRD results of the obtained LMOs is presented in Figure 3.

As the XRD results show (Figure 3), the reactions of the interaction of manganese oxide with lithium hydroxide took place at all durations. The XRD diffractogram of the sample obtained after exposure for 8 h showed the formation of the orthorhombic LiMnO_2_ phase, as well as the presence of a small amount of Li_0.4_MnO_2_, most likely reflecting the intermediate process of the formation of the main LiMnO_2_ phase. However, there was a phase of manganese oxide Mn_3_O_4_ that did not completely react with lithium hydroxide with a duration of 16 h. In addition, the spinel phase Li_0.78_Mn_1.88_O_4_ appeared, indicating the beginning of the manganese oxidation process. The diffractogram of the product of the process with a duration of 20 h also showed, along with the LiMnO_2_ phase, a lithium-manganese precursor with the spinel phase LiMn_2_O_4_, which can be represented as Li[Mn(III)Mn(IV)]O_4_.

All samples contain the LMO phases of various compositions, except for the sample obtained with a 13 h process. It was characterized by a monophase LiMnO_2_ that is most preferable for further synthesis of the sorbent. Therefore, the duration of 13 h was sufficient to pass the reactions of LMO formation.

Thus, the following conditions may be acceptable for the preparation of lithium-manganese oxides: temperature 125 °C, duration 13 h, the mass ratio of manganese oxides to lithium hydroxide monohydrate being based on the calculation to maintain the molar ratio Li/Mn = 1.

#### 3.1.2. Obtaining Precursors

The diffractograms of Figure 1, Figure 2 and Figure 3 are characterized by a very high background, indicating the presence of an amorphous component or insufficiently crystallized phase in the sample.

Various types of lithium-manganese spinels are promising precursors for obtaining sorbents for lithium extraction. Currently, there are only a few precursors for the production of sorbents or lithium-ion sieves (LIS) characterized by high lithium capacity, such as LiMn_2_O_4_, Li_4_Mn_5_O_12_, and Li_1.6_Mn_1.6_O_4_ [37]. LIS with one of the highest capacities was obtained from the Li_1.6_Mn_1.6_O_4_ precursor with a cubic structure by calcination from LMO with an orthorhombic structure of the LiMnO_2_ composition. In this case, the oxidation process for manganese from trivalent in the composition of LiMnO_2_ to tetravalent was required to obtain a precursor with the composition Li_1.6_Mn_1.6_O_4_.

Therefore, in order to obtain a lithium-manganese precursor with a sufficiently stable crystal structure, the next stage was calcination of the first-stage LMO. A batch of lithium-manganese oxide of the first stage was previously developed under the above selected conditions.

**Figure 4:**
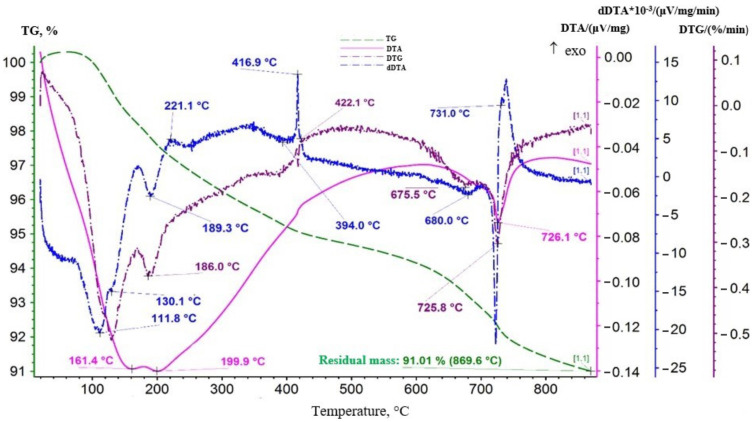
Thermogram of lithium-manganese oxide sample of the first stage of processing.

Before studying the calcination temperature, a sample of the produced batch of lithium-manganese oxide of the first stage was researched with the use of a thermal analysis method. The thermal analysis results for the sample are presented in Figure 4.

As it can be seen from Figure 4, the DTA curve showed endothermic effects of varying intensity with maximum development at 161.4, 199.9, and 726.1 °C. Additional effects were recorded on the dDTA curve. Their extremes were at 111.8, 130.1, 394, 680, and 731 °C. Additionally, exothermic peaks at 221.1 and 416.9 °C can be noted on the dDTA curve. All endothermic effects were developed against the background of a permanent decrease in the mass of the sample demonstrated by the TG curve course. The DTG curve formed a not very obvious maximum at 422.1 °C in the area of development of the exothermic effect (416.9 °C), and then a slight rise was observed. It indicated the occurrence of an oxidative process, i.e., on the oxidation of manganese (III) to manganese (IV), which was part of the lithium-manganese oxide LiMnO_2._ Effects in the temperature range of 100–200 °C were associated with the dehydration process. Adsorbed moisture was removed. The endothermic effect with an extremum at 726.1 °C on the DTA curve presumably reflected the decomposition of MnO_2_ with the release of oxygen. According to standards, the reaction occurred in the range of 600–700 °C. Perhaps the presence of lithium in the oxide effected the shift of the extremum toward higher temperatures. The combination of an endothermic effect with an extremum at 189.3 °C and an exothermic peak at 221.1 °C on the dDTA curve can be interpreted as a manifestation of an admixture of manganese dioxide gel.

**Figure 5:**
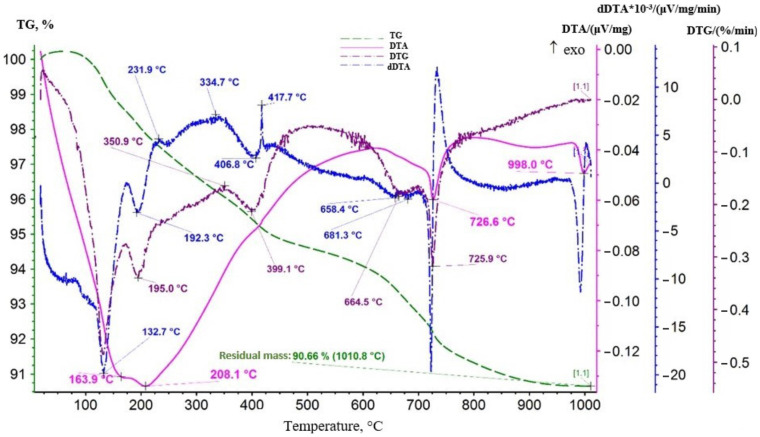
Thermogram obtained by repeated measurement of the lithium-manganese oxide sample.

A repeat measurement was performed to obtain additional information. The sample was increased to 0.4 g, and the heating interval was extended. The results of the study are presented in Figure 5.

**Figure 6:**
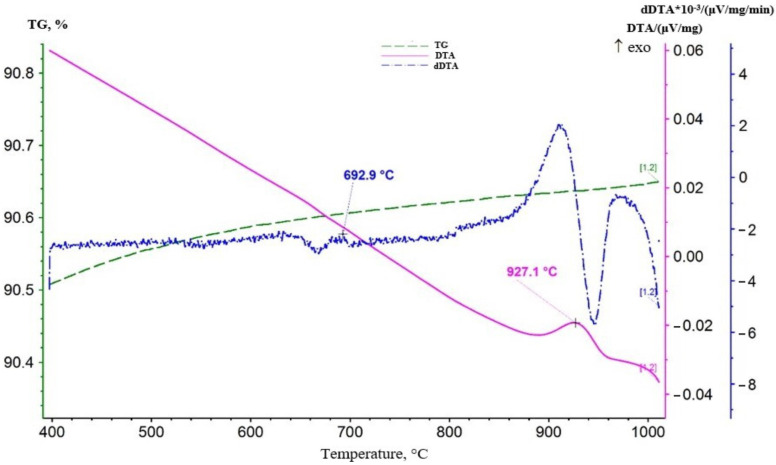
Plot of thermogram obtained during cooling of lithium-manganese oxide sample.

As it can be seen from the thermogram in Figure 5, an additional endothermic effect appeared on the DTA curve in this measurement with the maximum development at 998 °C. This effect was not accompanied by a change in mass, and on the DTA curve obtained during sample cooling (Figure 6) it corresponded to an exothermic peak at 927.1 °C.

In general, presumably, it is an enantiotropic polymorphic transformation of hausmanite—α-Mn_3_O_4_ (Mn^4+^Mn_2_^2+^O_4_) → β-Mn_3_O_4_. The dDTA curve showed a more clearly exothermic peak at 334.7 °C, accompanied by an increase in the mass of the sample, as indicated by the maximum at 417.7 °C in the DTG curve. For example, oxidative processes also occurred in the area of development of these effects. The combination of an endothermic effect with an extremum at 394 °C on the dDTA curve and an endothermic effect with maximum development at 998 °C on the DTA curve can presumably be interpreted as a manifestation of manganite—MnOOH. 

It is possible that the effect of lithium affected the shift in effect temperatures toward lower values. Thus, it can be assumed that the endothermic effect with extremum at 658.4 °C or 681.3 °C on the dDTA curve reflected the decomposition of lithium-manganese oxide—LiMnO_2_. Probably β-LiMn_2_O_3_ was formed.

According to the standards, the decomposition of β-kurnakite occurred in the temperature range 900–1050 °C. In our case, this decomposition may reflect an endothermic effect with maximum development at 726.6 °C. As a result, β-Mn_3_O_4_ (hausmanite) was formed.

The last endothermic effect with maximum development at 998 °C reflected the enantiotropic polymorphic transformation of hausmanite—β-Mn_3_O_4_ (Mn^4+^Mn_2_^2+^O_4_) → γ-Mn_3_O_4_. It was also impossible to exclude the possibility of transformation in the area of development of this effect α-Mn_3_O_4_ (Mn^4+^Mn_2_^2+^O_4_) → β-Mn_3_O_4_.

As the results of thermal analysis show, for the calcination process of lithium-manganese oxide LiMnO_2_ with an orthorhombic crystal lattice structure to form the cubic form Li_1.6_Mn_1.6_O_4_, it was necessary to study the calcination process of lithium-manganese oxide obtained at stage 1 in the temperature range from 350 to 600 °C, within which the oxidation processes of manganese present in the LMO should take place, from degree +3 to degree +4.

The effect of temperature and duration of calcination of lithium-manganese oxide was studied.

**Figure 7:**
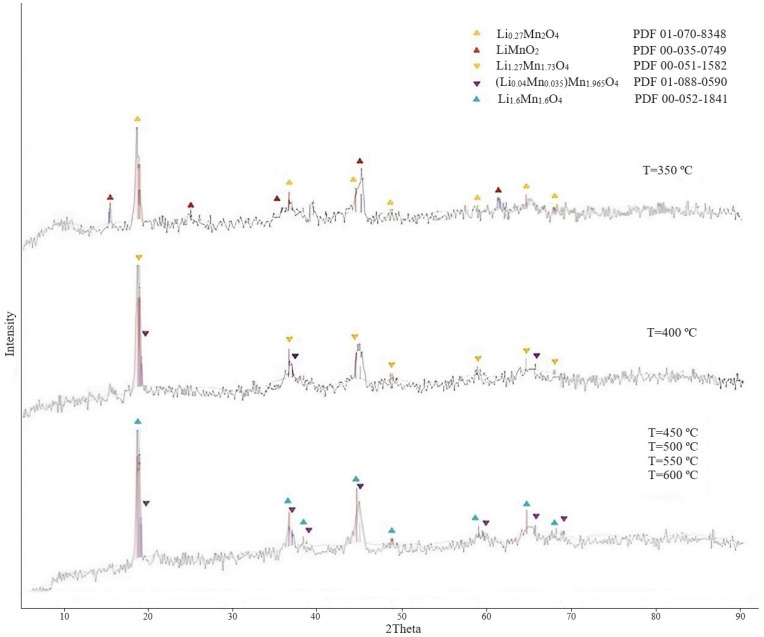
Diffractograms of lithium-manganese precursors obtained at different temperature exposures.

Effect of calcination temperature. The experiments were conducted under the following conditions: temperature 350, 400, 450, 500, 550, 600 °C; duration 5 h. Samples of the obtained precursors were investigated using X-ray phase analysis. The results of XRD are presented in Figure 7. The diffractogram of the sample obtained at a temperature of 350 °C indicated that the process was at the initial stage, since there was mainly a completely unformed phase of the composition Li_0.27_Mn_2_O_4_; there was also a residual phase of orthorhombic LiMnO_2_. At a temperature of 400 °C, the diffractogram was characterized by the formation of a phase of a lithium-manganese precursor of the composition Li_1.27_Mn_1.73_O_4_. It is clear from Figure 7 that the Li_1.6_Mn_1.6_O_4_ precursor phase was formed when LMO was calcined at temperatures from 450 to 600 °C. The authors of [38] came to the conclusion that, as the temperature decreased from 550 to 450 °C, the adsorption of lithium increased and the higher the temperature of precursor calcination, the worse the extractability of lithium from the precursor during sorbent preparation.

The most preferable calcination temperature is 450 °C based on the obtained research results.

**Figure 8:**
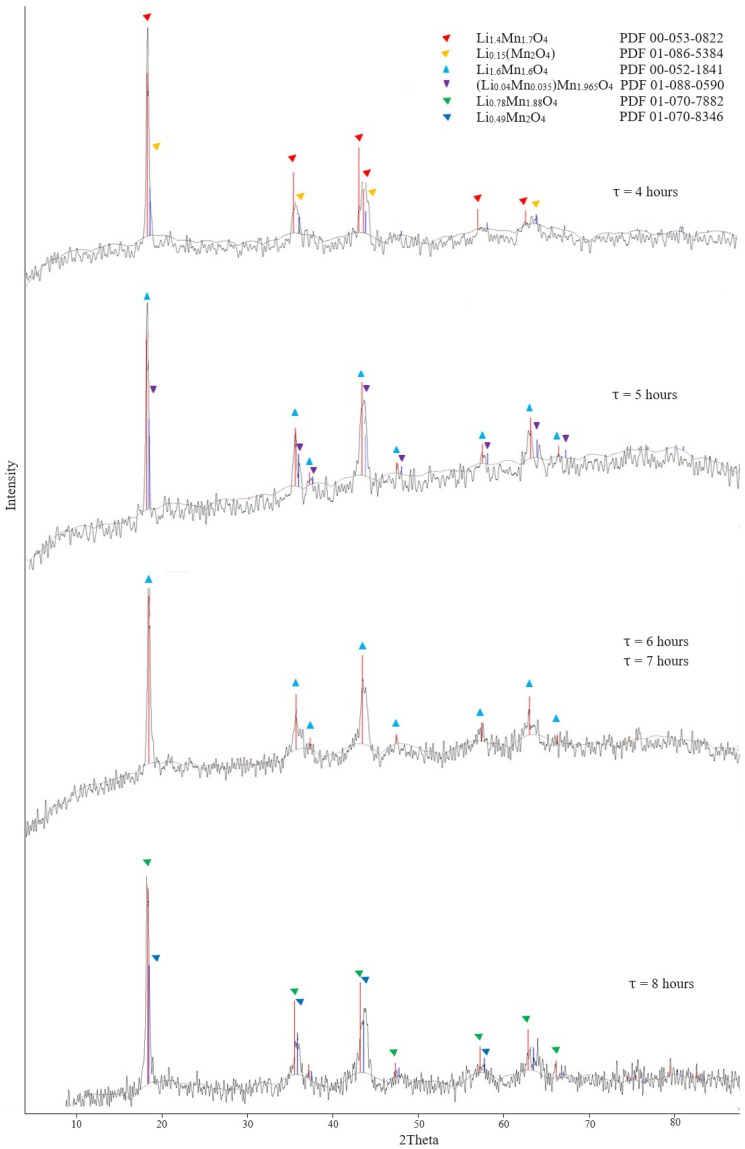
Diffractograms of lithium-manganese precursors obtained at different dwell times.

The effect of the duration of calcination was carried out under the following conditions: temperature 450 °C; duration 4, 5, 6, 7, and 8 h. The XRD patterns of the obtained precursor samples are presented in Figure 8.

The XRD data of the precursors presented in Figure 8 showed that lithium-manganese oxides Li_1.4_Mn_1.7_O_4_ were already formed at 4 h. However, the presence of phase Li_0.15_(Mn_2_O_4_) indicated that the process was not completed and additional time was required for the formation of precursor. The formation of the Li_1.6_Mn_1.6_O_4_ phase occurred upon exposure for 5 h. X-ray phase analysis of the precursor obtained after calcination at 450 °C for 6 h identified the Li_1.6_Mn_1.6_O_4_ monophase with a cubic structure in the sample; it can also be noted that the background of the diffractogram was significantly lower. It indicated good crystallization of the sample substance and a decrease in the amorphous component. 

The most preferable calcination conditions are a temperature of 450 °C and a duration of 6 h, according to the results of the studies.

### 3.2. Study of Acid Treatment Conditions for Lithium-Manganese Precursors

#### Acid Treatment of Lithium-Manganese Precursors

Acid treatment was performed to remove lithium from the lithium-manganese precursor and obtain a sorbent. Free vacant cells must remain during the removal of lithium from the precursor and, at the same time, in the structure of the resulting sorbent. They are very small in size and can only be occupied by lithium during sorption, or the replacement of lithium with a hydrogen atom that can be exchanged for a lithium atom during sorption. 

The effect of temperature, the ratio of precursor mass to acid volume, and duration on the acid treatment was studied. 

The effect of the process temperature was studied under the following conditions: temperature 30, 40, 50, 60 °C; HCl concentration 0.5 M; duration 12 h; the ratio of the sorbent mass to the acid solution volume of acid solution (S:L) = 1:800. The research results are shown in Table 1.

The research results obtained show that the extraction of lithium into the solution increased with an increase in the process temperature; and the extraction of lithium reached above 91% at 40 °C. Manganese losses over the entire temperature range studied were ~12.5–14%. The most preferable temperature is 40 °C, at which lithium extraction is 91%, manganese losses are 12.95%.

Study of the effect of the ratio of the precursor mass to the volume of the acid. The studies were conducted under the following conditions: temperature 40 °C; HCl concentration 0.5 M; duration 12 h; ratio of sorbent mass to acid solution volume (S:L) = 1:600; 1:700; 1:800; 1:900. The research results are shown in Table 2.

The experimental results show that lithium extraction was the maximum and amounted to ~90–91% while manganese losses were in the range from 12.28 to 12.95% at S:L ratios of S:L 1:700 and 1:800.

The effect of acid treatment duration was studied under the following conditions: temperature 40 °C; HCl concentration 0.5 M; S:L ratio = 1:800; duration 2, 6, 12, 18, and 24 h. The obtained results are shown in Table 3.

An increase in the acid treatment duration resulted in an increase in the transition degree of lithium into solution. The extraction reached ~90% or more at 12 h or more, while the loss of manganese remained practically unchanged throughout the studied duration of the process. The most preferable duration is 24 h, which makes it possible to achieve lithium recovery above 93%, under the data obtained.

**Figure 9:**
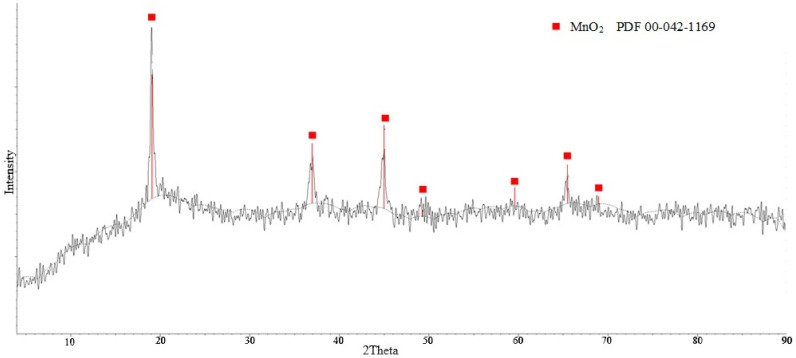
Diffractogram of the obtained sorbent.

X-ray phase analysis of the obtained sorbent presented in Figure 9 shows that it consisted of manganese dioxide monophase with cubic crystal lattice structure.

**Figure 10:**
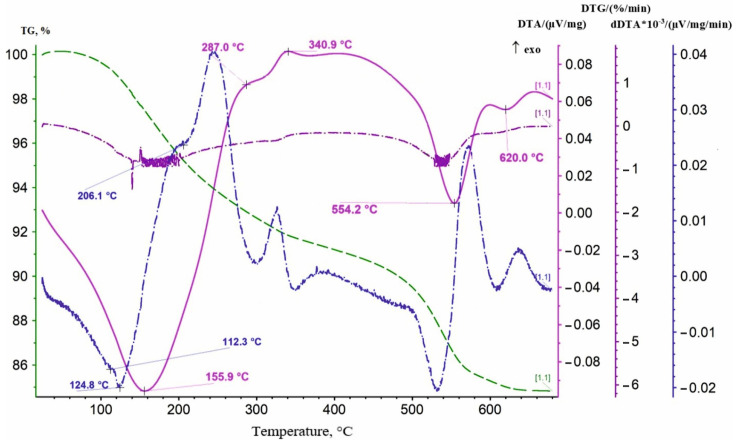
Thermogram of a sample of the obtained sorbent manganese dioxide.

A thermal analysis of the sample was conducted to clarify the composition of the resulting sorbent (Figure 10). The DTA curve showed endothermic effects of varying intensity with maximum development at 155.9, 554.2 and 620 °C. The most intense endothermic effect at 155.9 °C reflected the removal of chemically bound water, the protons of which can participate in the sorption process. The following two endothermic effects at 554.2 and 620 °C were possibly a manifestation of the decomposition of ß-MnO_2_ with the formation of ß-Mn_2_O_3._ The water content in the samples was determined by the weight loss during heating the sorbent sample at 450 °C. The H_2_O/Mn molar ratio was close to 0.5. The composition of the resulting sorbent apparently corresponded to the formula MnO_2_∙0.5H_2_O.

Thus, the study results of acid treatment showed that the most acceptable conditions for the process are temperature 40 °C, HCl concentration 0.5 M; S:L ratio = 1:700 and 1:800 and duration 24 h. In this case, the lithium extraction into the solution from the precursor can reach ~93%, and the lithium content in the sorbent is 0.277%.

### 3.3. Study of the Sorption Characteristics of the Obtained Sorbents

#### Study of the Process Conditions on the Lithium Sorption Recovery Characteristics

The sorption capacity of the sorbent increased with the increase in the pH value of the initial brine under the literature data [10]. Therefore, it was of interest to study the sorption capacity of the obtained sorbent at different pH of the initial brine.

Effect of the initial brine pH. The studies were at the following sorption conditions: temperature 35 °C, duration 24 h, ratio of sorbent mass to brine volume—1:6000; brine pH—7.32; 8.08; 9.08; 10.04; 11.08, and 12.06. The brine with the appropriate pH was prepared by adding a concentrated NaOH solution to the original brine with a pH of 7.32. Precipitates formed from the brine were filtered off. The initial brines and solutions after sorption were analyzed for the content of the studied components. The research results are presented in Table 4 and Table 5.

As can be seen from Table 4, the capacity of the sorbent for lithium increased, reaching a maximum capacity of 21.204 mg of lithium per 1 g of sorbent, at pH 12.06 with an increase in the pH of the medium and with the exception of pH 11.08. The capacity indicators of the synthesized sorbents based on manganese oxide obtained by various researchers have different values. In [36], a synthesized sorbent based on manganese oxide during the lithium sorption from geothermal fluid of Lumpur Sidoarjo (Lusi) showed a maximum capacity of 6.6 mg/g. In another work [10], the capacity of the synthesized sorbent H_1.6_Mn_1.6_O_4_ at the lithium sorption from brine was 22–27 mg/g, and a higher capacity of 34–40 mg/g was shown by the sorbent H_1.6_Mn_1.6_O_4_ at the lithium sorption from seawater [33].

Along with the capacity, the values of the distribution and separation coefficients during the sorption of lithium from brines by the synthesized sorbents are of great interest. As the calculated values of the distribution and partition coefficients presented in Table 5 show, lithium has the highest sorbent distribution values, and calcium—to a much lesser extent. In most cases, magnesium, sodium, and potassium are practically not sorbed on the manganese dioxide sorbent; and accordingly, the degree of separation of these metals from lithium is the maximum in all these cases.

For the initial brine with pH 7.32, the distribution coefficient for lithium was quite good, the separation of lithium from impurity macrocomponents also occurred at a fairly acceptable level.

Study of the effect of the ratio of sorbent mass to brine volume. The studies were performed at the following sorption conditions: temperature 35 °C, duration 24 h, pH of the initial brine 7.32. Varying the sorbent mass to the volume of the brine was performed at the following ratios—1:650, 1:1000, 1:2000 and 1:3000.

The sorption equilibrium characteristics during the extraction of lithium from brines with the use of a manganese dioxide sorbent were studied. The research results are presented in Table 6.

As can be seen from Table 6, the highest lithium extraction rates onto the sorbent occurred at a ratio of sorbent mass to brine volume of 1:650 and amounted to 85.9%.

The process duration effect was studied at the following conditions: temperature 35 °C, duration 6, 12, 24, 48 h, ratio of sorbent mass to brine volume—1:650. The research results are presented in Table 7.

The research results show that the sorbent recovery of lithium increased from 78.3 to 86.1% with an increase in the sorption duration from 8 to 48 h. As it can be seen from Table 7, the sorption process reached ~86% of lithium extraction onto the sorbent at the studied process conditions and a duration of 24 h. 

### 3.4. Determination of the Kinetic Model of the Lithium Sorption Process

#### Study of the Lithium Sorption Kinetics on a Synthesized Manganese Oxide Inorganic Sorbent

The study of the duration effect on the characteristics of the lithium sorption on synthesized manganese dioxide showed that the process takes a sufficiently long time to ensure acceptable extraction of the target metal from the brine onto the sorbent.

It is required to study the kinetics of the process in order to more thoroughly complete the sorption extraction of lithium from brines and optimize the process. Kinetic parameters can be useful to predict sorption rates and can also provide important information for the design and modeling of the sorption processes. Sorption is a complex and multistage process, and it is necessary to evaluate the adequacy of several kinetic models to identify the limiting stage.

Four kinetic models were used in the studies to analyze the kinetics of lithium sorption: pseudo-first and pseudo-second order models, the Elovich model, and the intraparticle diffusion model.

Brine with a Li concentration of 6.32 mg/dm^3^ with pH 7.32 at temperatures of 25 and 35 °C were used during kinetic studies for lithium sorption.

The linear form of the pseudo-first order model (Lagergren model) can be represented by the following equation [39]:

(6) $$\log\left(q_{e}-q_{t}\right)=logq_{e}-\left(\frac{k_{1}}{2.303}\right)t$$

where q_t_ and q_e_ (mg/g) are the number of lithium ions sorbed at time t (min) and at equilibrium with one g of sorbent, respectively, and k_1_ is the adsorption rate constant (1/min).

The Lagergren equation describes the patterns of sorption at the initial stages of the sorption process when the phenomenon of film diffusion has a significant effect on the process [40].

**Figure 11:**
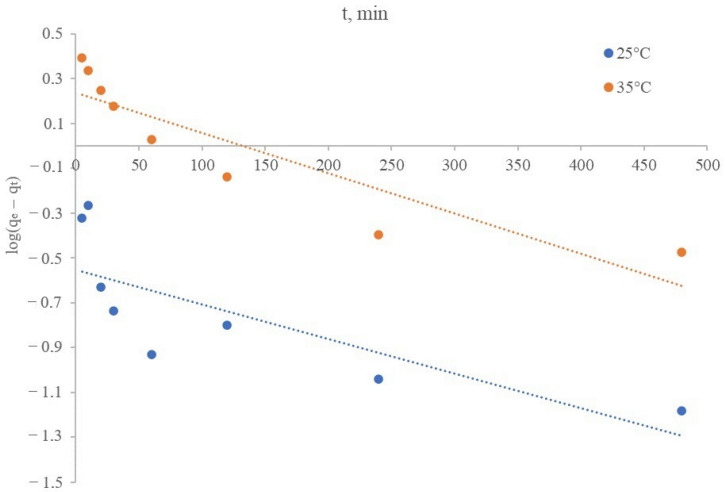
Description of the lithium sorption kinetics from brine in the coordinates of the pseudo-first order model.

The linear dependence presented in the log(q_e_ − q_t_) coordinates on t is shown in Figure 11, which describes the lithium sorption process from brine by a synthesized inorganic sorbent of manganese dioxide in accordance with Lagergren’s pseudo-first order model at temperatures of 25 and 35 °C.

The rate constant k_1_ can be determined experimentally by plotting log(q_e_ − q_t_) versus t.

Table 8 presents the kinetic parameters of the sorption process calculated from the data of the linear dependence log(q_e_ − q_t_) − t and Equation (6).

The values of sorption capacities q_e_ found from the graphical plots in Figure 11 for the process temperatures of 25 and 35 °C were 0.28 and 1.73 mg/g, and the correlation coefficients (R^2^) were 0.617 and 0.812, respectively. The data obtained on the lithium sorbent sorption, both at temperatures of 25 and 35 °C, did not fit into the pseudo-first order model.

As it can be seen from the calculated data (Table 8), the sorbent sorption capacity and the extraction, respectively, increased significantly with an increase in temperature. The dependence of the process on the temperature may indicate the chemical nature of the process rate limitations. 

The integral form of the classical pseudo-second-order velocity equation of Ho and Mackay has the following form [41,42]:

(7) $$q_{t}=\frac{t}{\frac{1}{k_{2}\cdot \;q_{e}^{2}}+\frac{t}{q_{e}}}$$

where k_2_—sorption rate constant of the pseudo-second order model, g/(mg∙min); t—time, min.

The equation was used in the following transformed form to process the experimental data:

(8) $$\frac{t}{q_{t}}=\frac{1}{k_{2}q_{e}^{2}}+\left(\frac{1}{q_{e}}\right)t$$

**Figure 12:**
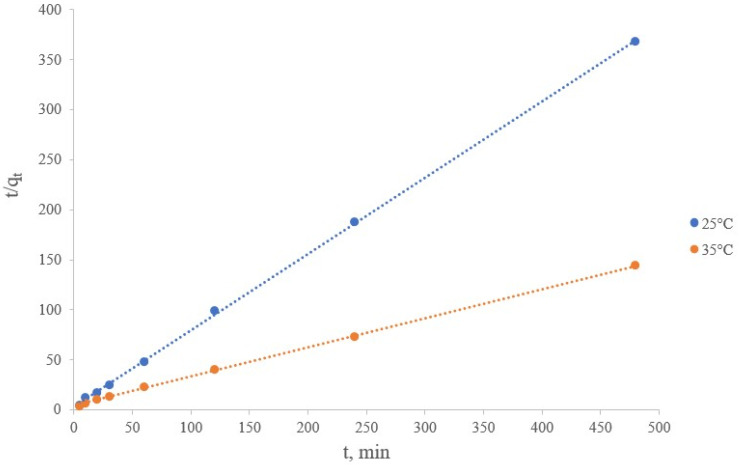
Description of the lithium sorption kinetics from brine in the coordinates of the pseudo-second order model.

The straight lines of the t plots $\frac{t}{q_{t}}$ shown in Figure 12 give the slope $\frac{1}{q_{e}}$ and intercept $\frac{1}{k_{2}q_{e}^{2}}$.

The results of graphical constructions and calculations presented in Table 8 show good comparability with the experimental data and suggest the applicability of the pseudo-second order kinetic model.

The calculated values of q_e_ were found to be 1.31 and 3.44 mg/g with the correlation coefficients (R^2^) of 0.9997 and 0.9995 at the process temperatures of 25 and 35 °C, respectively. The obtained data were approximated by a pseudo-second order model and were ideally close to the experimental results.

In accordance with the results obtained, the pseudo-second-order model is the most suitable for the description of the kinetic process of the lithium sorption on the synthesized manganese sorbent. It suggested that the kinetic process is mainly controlled by chemical sorption or chemisorption with the participation of valence forces due to the exchange of electrons between the sorbent and the sorbate [43]. Data from physical research methods of the sorbent indicate that it has a composition of MnO_2_∙0.5H_2_O.

Apparently, the sorption process occurred due to ion exchange between the lithium ion from the brine and the hydrogen proton that is part of the sorbent water molecule. A sorbent of a similar composition was obtained from its predecessor in accordance with studies [44], i.e., from the lithium-manganese precursor Li_1.6_Mn_1.6_O_4_. During the synthesis of manganese oxide sorbent under selected optimal conditions, its lithium-manganese precursor had a similar formula (Figure 8).

The Elovich kinetic model describes cases of heteroganic chemisorption on solid surfaces [45], i.e., applicable to the process of chemisorption between lithium ions and active proton-containing sites of the sorbent. The Elovich equation takes the contribution to the kinetics of adsorption and desorption processes into account. The linear form of the Elovich model can be represented by the following equation:

(9) $$q_{t}=\frac{1}{\beta }\ln\left(\alpha \beta \right)+\frac{1}{\beta }ln(t)$$

where α is the initial sorption rate (mg/(g∙min)), and β is the desorption constant (g/mg).

**Figure 13:**
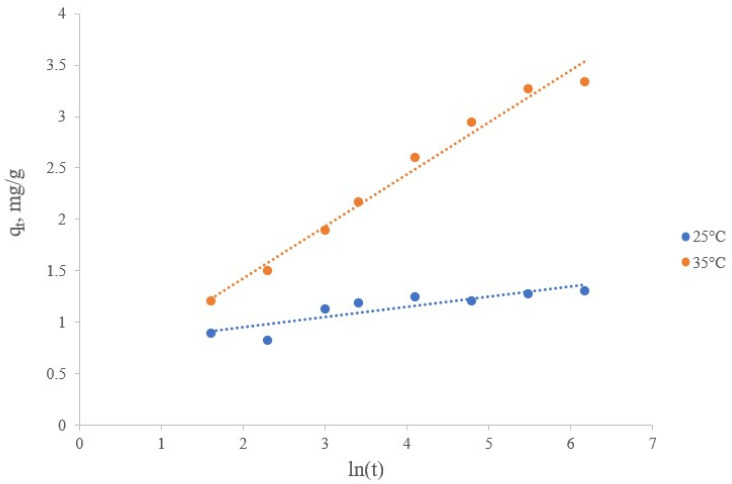
Description of the lithium sorption kinetics from brine in the coordinates of the Elovich model.

The values of the Elovich parameters are calculated from the slope and intersection of linear graphs of qt versus ln(t) (Figure 13 and Table 8).

The α values for the sorption of lithium ions on the manganese sorbent decreased from 203.625 to 1.163 mg/(g∙min), and the β values also decreased from 10.111 to 1.982 g/mg at 25 and 35 °C of the sorption process, and respectively, the values of the correlation coefficient (R^2^) were 0.7604 and 0.9818, respectively. The data obtained confirmed that the Elovich model does not agree with the experimental data, which primarily concerns the process at 25 °C. According to the calculated values, the process with a lower temperature of 25 °C was characterized by a very high initial rate of sorption and desorption, while with an increase by 10 °C the initial rate of sorption and desorption decreased significantly.

On the other hand, the sorption of lithium ions from brines on a manganese dioxide sorbent can be represented as a multi-stage process. The first stage includes the transport of lithium ions from the bulk of the brine to the solid surface of inorganic sorbent particles, characterized by volumetric diffusion. Then, the second stage occurs by the diffusion of lithium ions into the boundary layer of solid manganese dioxide sorbent particles, considered as film diffusion. This is followed by a third stage where lithium ions are transported from the surface to the internal pores (pore diffusion or intraparticle diffusion). The last stage will likely be a slow process. The intraparticle diffusion model can be applied using the Weber and Morris equation [46]:

(10) $$q_{t}=K_{id}t^{0.5}+C$$

where q_t_ (mg/g) is the amount of lithium sorbed at time t, K_id_ (mg/(g min 0.5)) is the rate constant of intraparticle diffusion, C is the thickness of the boundary layer.

**Figure 14:**
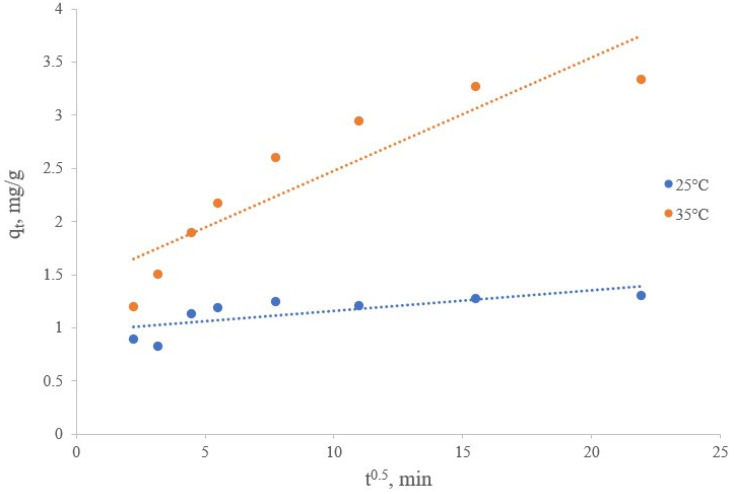
Description of the lithium sorption kinetics from brine using the intraparticle diffusion model.

The linear dependence, presented in the q_t_ coordinates from t^0.5^, describing the lithium sorption process from brine by a synthesized inorganic sorbent of manganese dioxide under the Weber and Morris model of intraparticle diffusion at temperatures of 25 and 35 °C, is shown in Figure 14. Intraparticle diffusion parameters were calculated from the slope and intercept of line graphs, as shown in Figure 14. Graphs of q_t_ versus t^0.5^ show that the resulting straight lines did not pass through the origin (C > 0). It was found according to the data obtained as shown in Table 8 that the values of the correlation coefficient R^2^ were 0.5516 and 0.8290, the intraparticle diffusion rate constants K_id_ were 0.0194 and 0.1068 mg/(g∙min^0.5^), and the thickness of the boundary layer C was 0.9625 and 1.4125 mg/g at sorption process temperatures of 25 and 35 °C, respectively. The data obtained confirmed the unsuitability of this model for description of the sorption kinetics.

Based on the kinetic parameters of the four kinetic models, as seen in Table 8, the sorption kinetics were estimated and are in good agreement with the pseudo-second order kinetic model.

Thus, the data obtained show that the process of the lithium sorption from brines on a synthesized inorganic sorbent of manganese dioxide could be described by the Ho and Mackay pseudo-second order equation, and that chemical kinetics occurred. The kinetic parameters were significantly affected by the sorption conditions, in particular temperature and duration.

## 4. Conclusions

The obtained research results show that, at the first stage of sorbent preparation at the exposure of the mixture of lithium hydroxide and manganese oxides taken from the calculation of Li/Mn molar ratio maintenance = 1, at temperature 125 °C, duration of 13 h, the lithium-manganese oxide of LiMnO_2_ composition with orthorhombic structure is formed.

Thermal analysis showed that, in order to undergo the calcination process of lithium-manganese oxide LiMnO_2_ with an orthorhombic crystal lattice structure until the formation of a cubic form Li_1.6_Mn_1.6_O_4_, calcination should preferably be carried out at least at a temperature of 450 °C, but not higher than 600 °C, within which the processes of oxidation of manganese present in the composition of LMO should take place, from degree +3 to degree +4. At temperatures above 600 ° C, the decomposition reaction of manganese dioxide can begin with the formation of oxides of lower valencies.

Acid treatment of the precursor 0.5 M HCl is preferably carried out under the following conditions: temperature 40–50 °C, HCl concentration 0.5 M; ratio S:L = 1:700 and 1:800, and duration 24 h. At the same time, the extraction of lithium into solution from the precursor can reach ~93–97%.

It is possible to recover lithium by ~86% from brine with a low content of the target component—5.9–7.8 mg/L lithium with the use of resulting sorbent.

A kinetic model of the lithium sorption process was determined. The adequacy of several kinetic models was assessed to identify the rate-limiting stage.

Four kinetic models were used in the studies to analyze the kinetics of lithium sorption—pseudo-first and pseudo-second order models, the Elovich model, and the intraparticle diffusion model. According to the research results, the pseudo-second-order model is the most suitable for description of the lithium sorption process kinetics on a synthesized manganese sorbent and assumes that the chemical exchange reaction limits the process.

## Figures and Tables

**Table 1:** The effect of temperature on the acid treatment of the precursor.

Temperature, °C	Lithium Content in the Sorbent, %	Lithium Extraction into Solution, %	Loss of Manganese in Solution, %
30	1.698	83.77	12.51
40	1.041	91.02	12.95
50	1.003	97.49	13.38
60	0.692	98.00	13.94

**Table 2:** The effect of the S:L ratio on the acid treatment of the precursor.

Ratio S:L	Lithium Content in the Sorbent, %	Lithium Extraction into Solution, %	Loss of Manganese in Solution, %
1:600	1.117	88.90	11.34
1:700	1.033	90.38	12.28
1:800	1.041	91.02	12.95
1:900	1.150	88.33	12.72

**Table 3:** Effect of process duration on acid treatment of precursor.

Duration, h	Lithium Content in the Sorbent, %	Lithium Extraction into Solution, %	Loss of Manganese in Solution, %
2	2.095	75.58	12.35
6	1.693	81.55	12.31
12	1.041	91.02	12.95
18	0.332	89.13	12.51
24	0.277	93.26	12.91

**Table 4:** Compositions of initial brines and sorbent capacity for lithium.

pH of Initial Brine	Concentration in Initial Brine, g/L					SEC, mg/g
	Li, mg/L	Ca	Mg	Na	K	
7.32	6.389	2.329	0.759	19.74	0.376	10.00
8.08	8.094	2.204	0.721	17.88	0.348	9.39
9.08	8.392	2.317	0.756	18.83	0.449	12.39
10.04	8.129	2.298	0.690	18.59	0.408	18.948
11.08	7.838	2.185	0.032	21.45	0.387	12.408
12.06	7.661	2.047	0.001	21.86	0.373	21.204

**Table 5:** Distribution and partition coefficients for the lithium sorption from brine depending on the pH.

pH of Initial Brine	Distribution Coefficient, K_d_					Partition Coefficient, K_s_			
	Li	Ca	Mg	Na	K	Li/Ca	Li/Mg	Li/Na	Li/K
7.32	2116	224	221	0	0	9.4	9.6	-	-
8.08	1438	71	67	0	0	20	21	-	-
9.08	1958	73	0	0	207	27	-	-	9.4
10.04	3811	0	0	0	0	-	-	-	-
11.08	2150	72	0	318	0	30	-	6.8	-
12.06	5138	110	0	398	0	46	-	13	-

**Table 6:** Effect of the ratio of sorbent mass to brine volume on the characteristics of sorption extraction of lithium from brine.

Ratio m_sorbent_ to V_solvent_	Lithium Recovery onto Sorbent, %	SEC, mg/g	Distribution Coefficient, K_d_
1:650	85.9	3.66	3952
1:1000	66.7	4.15	2003
1:2000	53.2	6.62	2273
1:3000	39.2	7.33	1937

**Table 7:** Effect of process duration on the characteristics of sorption extraction of lithium from brine.

Duration, h	Extraction of Lithium onto Sorbent, %	SEC, mg/g	Distribution Coefficient, K_d_
8	78.3	3.33	2340
16	83.7	3.57	3348
24	85.9	3.66	3952
48	86.1	3.67	4038

**Table 8:** Kinetic parameters of the lithium sorption by manganese dioxide sorbent.

Kinetic Model	Parameters	Temperature of the Sorption Process, °C	
		35	25
Pseudo-first order	q_e_, mg/g	0.28	1.73
	k_1_, 1/min	3.454 × 10^−3^	4.145 × 10^−3^
	R^2^	0.6175	0.8123
Pseudo-second order	q_e_, mg/g	1.31	3.44
	k_2_, g/(mg min)	0.170	0.019
	h, g/(mg min)	0.292	0.224
	R^2^	0.9997	0.9995
Elovich model	α, mg/(g min)	203.626	1.162
	β_t_, g/mg	10.111	1.982
	R^2^	0.7604	0.9818
Intraparticle diffusion model	k_id_, mg/(g min^1/2^)	0.0194	0.1068
	C, mg/g	0.9625	1.4125
	R^2^	0.5516	0.8290
Experimental sorbent capacity	q_exp._, mg/g	1.37	3.67

## Data Availability

The data and results presented in this study are available in the article.
